# The Structure of Ordered Mesoporous Materials Synthesized from Aluminum Phyllosilicate Clay (Bentonite)

**DOI:** 10.3390/molecules28062561

**Published:** 2023-03-11

**Authors:** Malgorzata Zienkiewicz-Strzalka, Stanislaw Pikus, Malgorzata Skibinska, Magdalena Blachnio, Anna Derylo-Marczewska

**Affiliations:** Faculty of Chemistry, Maria Curie-Sklodowska University, M. Curie-Sklodowska Sq. 3, 20-031 Lublin, Poland

**Keywords:** mesoporous silica materials, mesoporous aluminosilicate composites, functional materials, SBA-15, bentonite

## Abstract

This paper reports the synthesis and structural analysis of mesoporous silica materials with the use of aluminum phyllosilicate clay (bentonite) as an alternative silica source. In the proposed synthesis, bentonite, as natural aluminosilicate, was used instead of commercially available and quite expensive tetraethyl orthosilicate (TEOS) silica source. The objective of the research study was to determine the effect of aluminum loading in the mesoporous silica body for ordering structure, porosity, and potential sorption capacity to thorium ions. The unique direction developed in this procedure is focused on preparing advanced materials from natural sources with their own desired functionality and general availability. The applied procedure based on the classic, one-step synthesis of SBA-15 silicates was modified by gradually increasing the bentonite amount with simultaneous reduction of the TEOS content. The structural and morphological characterization, as well as evaluation of the porous structure of the obtained materials, was performed using powder wide-angle X-ray diffraction (XRD), small-angle scattering (SAXS), transmission and scanning electron microscopy (TEM, SEM), low-temperature nitrogen adsorption–desorption methods and potentiometric titration. The new, cost-effective composites for the removal of Th(IV) ions are proposed. The synergistic effect of expanding the porous surface using bentonite as a silica precursor and the presence of thorium-binding groups (such as Al_2_O_3_) is indicated.

## 1. Introduction

The successful fabrication of ordered mesoporous silica materials (OMS) has been an important event in the development of materials science and engineering. It began when a new silicate material called MCM-41 (Mobil Composition of Matter N°41, M41S) was developed by scientists at Mobil Oil Company [1]. MCM-41 is characterized by a hexagonal arrangement of cylindrical mesopores with a narrow diameter distribution and a large specific surface. Despite amorphous pore framework walls, this material has a crystalline structure, which results from periodic arrays of the pores system [2,3]. Further investigations have shown that the length of surfactant macromolecule strongly affects the mesopores’ diameter and surface area development. In terms of morphology, spherical particles are the most common product of the alkaline process [4], and size of the particles is pH-dependent. For example, Varache et al. reported that an increase in NaOH concentration results in larger particles [5]. The same group of scientists prepared the MCM-48 and MCM-50 silicas, which are with MCM-41 members of the M41S family. Similar to the MCM-41 synthesis, the process is based on a templating using an ionic surfactant, silica precursor, and other organic compounds, e.g., methanol or 2-methylpropan-2-ol [6,7]. The mesophase forming proceeds in a slightly alkaline system. The silica source/surfactant ratio is the critical factor determining a type of porous structure. Preparation of the cubic (MCM-48) and lamellar structures (MCM-50) requires values of this ratio in the range of 0.65–1.5 or 0.5–0.83, respectively [8,9]. Due to the lamellar structure, MCM-50 shows very poor hydrothermal stability, and thus during the removal of the template, its pore walls collapse, resulting in a limited specific surface area of the final product. The MCM-50 quality can be improved by the appropriate selection of reagents and experimental parameters of their synthesis (raising the temperature and pH reaction system, extending reaction time, and using surfactants with loner alkyl chains and silica precursors at higher concentrations compared to those in the MCM-41 preparation procedure) [8].

Parallel to the changes in the structure of silica materials, there was a trend to change the chemical, physicochemical and textural properties. Since then, efforts have been made by the scientific community to better understand and utilize the supramolecular templating process and to develop new synthesis procedures for OMS. As an example, Tanev and Pinnavaia obtained the HMS (Hexagonal Mesoporous Silica) materials using various nonionic primary amine surfactants (C8 to C18) in water–ethanol solutions [10]. Silicas have greater textural mesoporosity, thicker walls, and better thermal stability compared to MCM-41. Finally, Zhao’s research group at Santa Barbara University developed a material called SBA-15 (Santa Barbara Amorphous N°15) [11]. The novelty of the synthesis procedure is the use of amphiphilic triblock copolymer and strong acidic conditions of the reaction system (pH < 1). Here, as in the processes described above, TEOS plays the role of the silica precursor. Most often, HCl is used as a catalyst, although other strong acids (H_2_SO_4_, HNO_3_, HI, HBr) are also used. Weak acids (CH_3_COOH, H_3_PO_4_) are not suitable due to the poor quality of the resultant materials [12,13]. Generally, the hydrothermal method of synthesis using non-ionic surfactant is applied [11,14], but there have also been reports on the microwave and ambient temperature methods which favor shortening the time and decreasing the temperature of the process, respectively [15,16]. As received, SBA-15 is characterized by an ordered hexagonal two-dimensional structure, a well-developed surface area, and the presence of micropores. Both the total pore volume and average pore diameter are larger than those for MCM-41 silica. The relatively large thickness of the pore walls makes this material hydrothermally and mechanically stable [17]. Thermal stability, various types of porosity with homogeneous characteristics (micropores and mesopores), as well as ease of synthesis and predictability of the structure, made this type of OSM system widely applicable. Thus, the appropriate selection of conditions of the SBA-15 synthesis, i.e., temperature and time of reaction system (at the aging stage), the ratio of reactants, acid concentration, the addition of cosurfactant organic molecules or swelling agent to the mother solution allows controlling the range of pore distribution and pore walls thickness [11,13,18,19,20,21].

Despite using a nonionic block copolymer, the synthesis of MSU (Michigan State University) is performed at neutral pH conditions. Here, the control of the formation of mesoporous ordered structures is provided by using fluoride catalysts [22,23]. The unconventional approach to the composition of the reaction mixture has been presented in the FSM-16 (Folded Sheet Materials) synthesis, where kanemite as a silica precursor is used [24]. It is a mineral with the formula NaHSiO_2_∙3H_2_O, whose structure consists of tetrahedral layers of SiO_4_ and sodium cations between them. At the beginning of synthesis, an exchange reaction between sodium cations and alkyltrimethylammonium ions derived from the surfactant takes place, followed by a condensation process of kanemite silicate layers. The final product shows a one-dimensional hexagonal arrangement of channels and a well-developed surface area.

So far, three models of forming the OMS materials have been proposed: a puckering layered model, silicate rod assembly, and cooperative charge density matching [25]. The last one assumes several types of organic–inorganic hybrid interfaces for mesostructure formation based on mutual interactions between ionic/nonionic surfactant (or nonionic copolymer), ionic oligomeric silica species (or/and hydroxyl groups) and counter-ions of an inorganic substrate (halide anions). Respective types are as follows: S^+^I^−^ (electrostatic interactions in the MCM-41, MCM-48, MCM-50 forming), S^+^X^−^I^+^(electrostatic interactions in the SBA-1, SBA-2, SBA-3 forming), N°I° (hydrogen-bonding force in the MSU forming), N°X^−^I^+^ (electrostatic interactions in the SBA-15 forming), S°I° (hydrogen-bonding force in the HMS forming), with S-surfactant species, N-nonionic copolymer species, I-inorganic silica species, X−counter-ions. This proves the mechanism of interactions in the reaction system is pH, and the reagents are type dependent.

Over the past two decades, the variety of morphological (spherical, rod-like, carambola-shaped, oval) and structural forms (hexagonal, regular, lamellar) of ordered mesoporous silica materials influenced by the type of synthesis method, reagents, and conditions has been observed. Additionally, specific surface properties were imparted to OMS by various modification processes, i.e., grafting, co-condensation, or creating hybrid core-shell structures. The enormous application potential of the obtained materials in sorption [26,27,28,29,30,31,32], catalysis [33,34,35,36], delivery [37,38,39,40,41,42,43], separation [44], chromatography [45], energy conversion, and storage [46,47,48] is a result of the development of new synthesis pathways. The examples shown above do not constitute an exhaustive list of applicant potential of OMS. This application list is broad and still open.

In this work, the green approach in the SBA-15 synthesis is presented. The proposed new strategy is based on using aluminum phyllosilicate (bentonite) as an alternative silica source to commercial agents such as TEOS, TMOS (tetramethyl orthosilicate), or TBOS (tetrabutyl orthosilicate) [49,50,51]. The solution proposed is in line with the current trends in the promotion of natural materials in chemistry and material engineering. Attempts of using renewable raw materials, such as rice husk, diatoms, energy wastes (coal ash), or photoelectric wastes (semiconductors) for the synthesis of ordered mesoporous materials, have been already reported [41]. The mineralogical and chemical composition of bentonite comprises mainly of silica (SiO_2_), alumina, (Al_2_O_3_), and iron oxide (Fe_2_O_3_), in the approximate amounts of 61 wt%, 18 wt%, and 2 wt%, respectively [52]. Although the composition is slightly different depending on the origin, generally, this clay seems to be a suitable reservoir of silica. Other advantages of this approach concern additional sorption sites related to alumina in the mineral as well as lower cost of production and availability in the world [52,53,54,55,56]. To confirm the usefulness of materials synthesized from aluminum phyllosilicate clay, the results of the study on the removal of Th(IV) ions from aqueous solutions were presented. The content described in this work was aimed at assessing the quality of new materials in which TEOS has been partially or completely replaced with bentonite. The research aimed to find a cheaper solution, combining ecological aspects and finding a compromise between the functionality of bentonite (which is a cheap adsorbent for water and wastewater treatment) and an ordered structure valuable for specific applications (when only TEOS is used). The choice of thorium as potential contamination was dictated by its numerous applications in the electrotechnical industry, which is associated with the production of wastes that pose a threat to the natural environment. In addition, the chemical similarity of thorium to other more toxic tetravalent actinides allows for treatment as a model substance. Ultimately, the superiority of the synthesized composites over mineral adsorbents (bentonite) resulting from both porous structure and a variety of adsorption moieties was verified.

## 2. Results

The following section presents the results obtained by attempting a new approach to the synthesis of mesoporous silica materials. By definition, such materials are characterized by an ordered structure. However, is it preserved with drastic component changes? What is the relationship between the porous structure and the SiO_2_ source? We will try to answer these questions using an attempt to synthesize silica materials with bentonite as a TEOS substitute or an accompanying substance.

### 2.1. Characterization of the Materials by X-ray Diffraction

**Figure 1:**
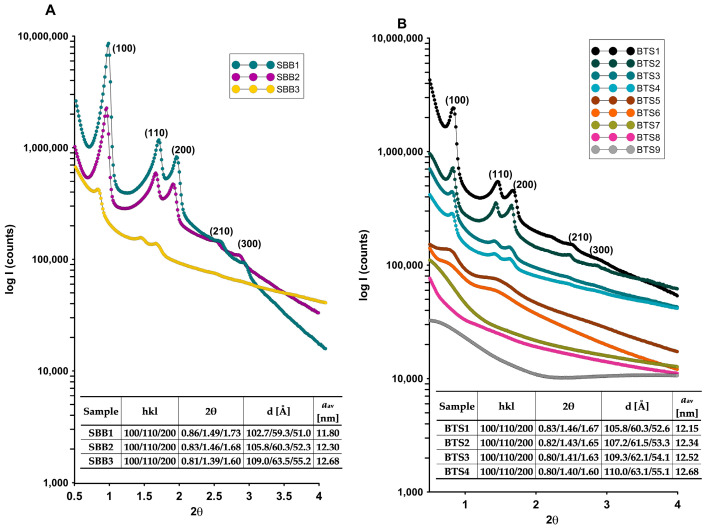
The SAXS patterns for the investigated composites: (**A**) SBB1–SBB3 and (**B**) BTS1–BTS9.

Conventional materials characterization for the SBA-15-like pore model (micro- and mesopores) requires a combination of analysis methods for the description of both the particle and the pore structure. The quality of the ordered structure of the synthesized mesoporous aluminosilicate composites was investigated by SAXS. A characteristic feature of a hexagonally ordered array of pores is the presence of diffraction peaks in the low angle range of the XRD curve. Figure 1 presents the experimental curves for the investigated samples. In the case of the samples synthesized with a standard amount of TEOS (Figure 1A), a hexagonal structure of SBA-15 is found. However, regardless of the bentonite amount, the hexagonal symmetry of the silica lattice is still visible (for three investigated samples), but their quality deteriorates for a higher concentration of the aluminosilicate (the same TEOS addition). One intense peak at 0.80 degrees of 2θ and two additional with lower intensities at 1.48 and 1.75 2θ degrees of (100), (110), and (200) lattice planes correspond to long-range structure ordering and a well-formed two-dimensional hexagonal lattice of the *p6mm* symmetry.

Based on the calculated unit cell parameter, it can be seen that it changes slightly depending on the amount of bentonite added and equals 11.80 nm, 12.13 nm, and 12.03 nm for SBB1, SBB2, and SBB3 samples, respectively. This indicates the increasing dimension of the unit cell with the increasing amount of bentonite and the significant impact of the growing silicon content in the synthesis mixture. One explanation of this phenomenon may be based on the gradual decomposition of the bentonite during the synthesis and the effect of additional silica amount in the process of the micellar matrix formation (responsible for their size). The above data clearly show that the synthesis using bentonite as a silica precursor leads to the formation of an ordered material when TEOS is also involved (SBB1–SBB3). Then, even a significant increase in the amount of bentonite results in obtaining a material with an ordered structure.

In the case of samples synthesized with decreasing amounts of TEOS and increasing amounts of bentonite (BTS1–BTS9), the well-defined hexagonal structure was observed only for the first four samples marked as BTS1–BTS4 (Figure 1B). Such changes in reaction mixture composition result in a gradual loss of structural ordering. The high quality of the mesoporous ordered system for BTS1 and BTS2 samples was reflected as even five peaks indexed as (100), (110), (200), (210), and (300) from hexagonal symmetry, and it seems to produce even better results than for typical (only TEOS) procedure [57].

The SAXS patterns of the BTS5 sample (2.15 g of TEOS and 3.45 g of bentonite) reveal a lack of specific signals and lifting of the scattering line instead of those characteristic peaks for the SBA-15 structure. The observation shows that the emerging phase is gradually changed at this specific amount of TEOS and bentonite. These data were cross-checked and confirmed by repeated synthesis. In the case of subsequent samples with further increasing of the bentonite amount and with decreasing of TEOS, structural ordering is lost, and the amorphous structure of the composites is formed (BTS5–BTS9).

The unit cell parameter increases with the growth of the bentonite amount and equals 12.15 nm, 12.34 nm, 12.52, and 12.68 nm for the BTS1, BTS2, BTS3, and BTS4 samples, respectively. Moreover, in all cases, the unit cell parameter is higher than for pure SBA-15 (12.0 nm) [58]. The successive increase in the lattice constant is associated with a growth in pore diameter observed above.

**Figure 2:**
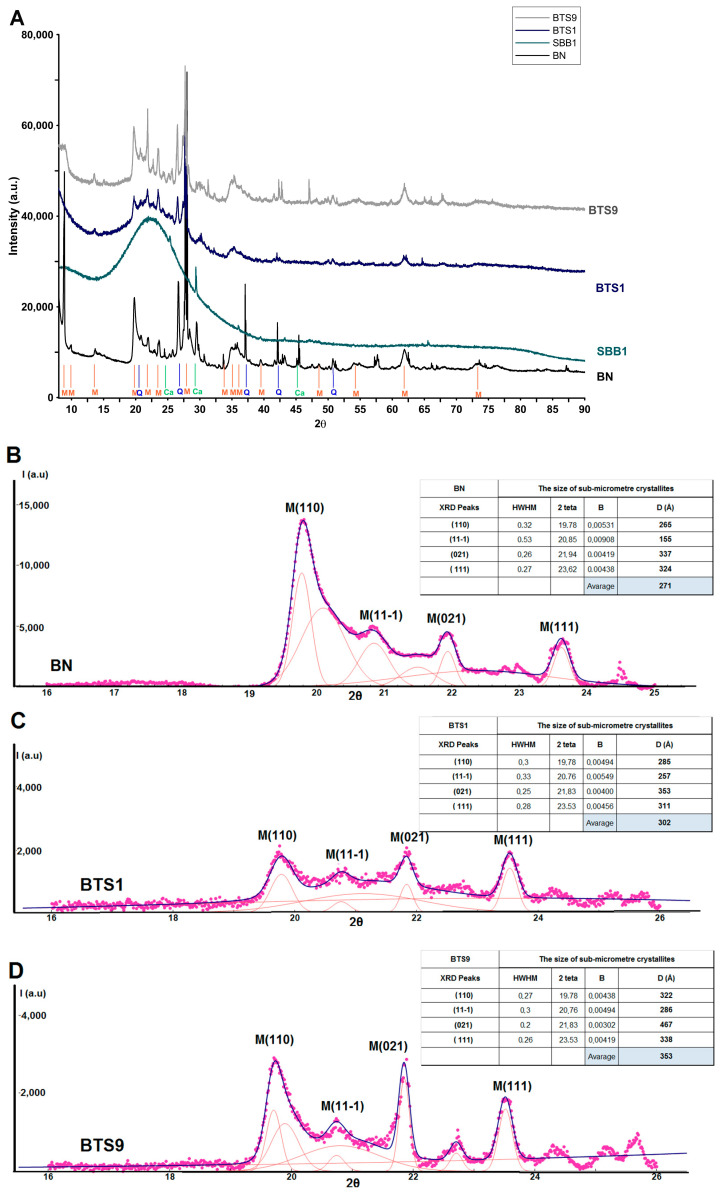
(**A**) The wide-angle XRD patterns for investigated materials: SBB1, BTS1, BTS9, and bentonite (BN). Phase analysis by XRD was referenced as montmorillonite (M), quartz (Q), and calcite (Ca), (**B**–**D**) deconvolution of diffraction peaks of montmorillonite phase as four first and presence peaks for analyzed phases ((110), (11-1), (021) and (111)) BN (**B**), BTS1 (**C**) and BTS9 (**D**).

The crystal structure of bentonite is reflected by diffraction patterns and suggests the presence of various types of minerals (Figure 2, black curve, marked as pure BN). However, the crystal structure of bentonite gets blurred when mesoporous aluminosilicates were created. The crystal structure of bentonite is almost invisible for all samples from the first group of composites (SBB1–SBB3) (from 0.5 to 1.5 g of bentonite). These materials do not exhibit significant long-range atomic order and mostly broad scattering peaks at 20 degrees of 2θ suggest the amorphous structure of silica. Only the traces of the crystalline form of bentonite are noticeable for this group of materials (Figure 2). The results of the composite prepared from bentonite are quite similar to the materials synthesized only from TEOS. Powder diffraction data at wide angles registered typically for SBA-15 materials do not show any diffraction peaks. It means that amorphous SBA-15 is not characterized by long periodicity in contrast to crystalline materials (such as bentonite with long-range order). In the case of mesoporous aluminosilicates, the atoms are mostly randomly distributed in 3D space, and it suggests the successful decomposition of the bentonite precursor in the synthesis mixture. Figure 2 shows also the wide-angle X-ray scattering profiles for two selected samples from the second group of composites (BTS1 and BTS9). The amorphous silica structure was observed next to the visible crystalline form of the mineral. For the rest of samples, the gradual changes in crystallinity were found. However, an initial analysis of the diffraction pattern confirms the lack of the peak at 9 degrees of 2θ for composites compared to pure bentonite, which originated from the layered structure of the mineral. This observation suggests that some parts of bentonite are not consumed during the synthesis process and may be transformed into a derivative structure. A comparison of two selected samples: BTS1 and BTS9 produced with decreasing/increasing amounts of TEOS/bentonite (Figure 2A) show that the intensity of visible diffraction peaks changed significantly with the increasing bentonite content.

In addition, the Scherrer equation was used to calculate the average crystallite size overwhelming the phyllosilicate phase (Montmorillonite). The nanocrystallite size D was calculated as a function of the peak width using the Scherrer equation: D = kλβcosθ, where k is a constant related to crystallite shape (k = 0.9), λ is the X-ray wavelength and equals 1.5418 Å, θ is the peak position in radians, and β is the full width at half maximum (in radians) of the peaks located at any 2θ position. Fitting of the experimental patterns was performed using the Fityk program. The crystallite size was determined from the full width at half maximum of the X-ray of four initial and nearby XRD peaks of (110), (11-1), (021), and (111) lattice planes of the monoclinic crystal form (C12/m1). The calculated values (from four values for each sample) show that the average size of the crystallites equals 271 Å, 302 Å, and 353 Å for the BN, BTS1, and BTS9 samples, respectively. A small amount of bentonite used as a co-precursor (BTS1) allows to obtain of a material in which the separated bentonite phase has larger dimensions than the initial precursor (302 Å instead of 271 Å for BN). Raising the amount of bentonite (for the BTS9 sample) increases the size of the phyllosilicate crystallites.

### 2.2. Characterization of the Materials by Gas Adsorption/Desorption Measurements

**Figure 3:**
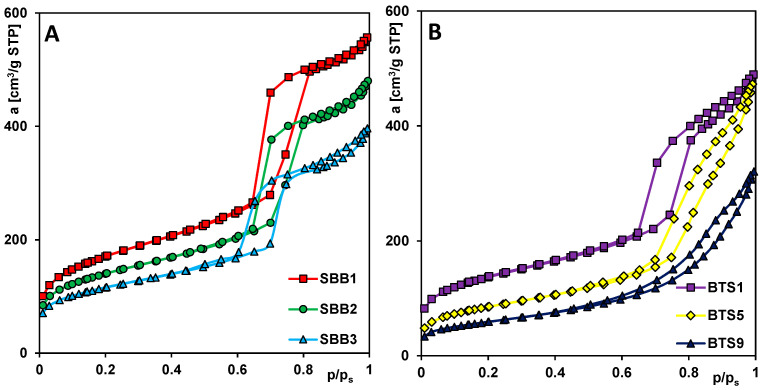
Nitrogen adsorption/desorption isotherms of selected samples: (**A**) SBB1-SBB3, (**B**) BTS1 and BTS9.

Insight into the size and the distribution of pores of composite material is very important to understand adsorption potential. The porous structure of the obtained materials was investigated by nitrogen low-temperature adsorption–desorption technique. The obtained adsorption–desorption data allow to determine the relations among the pore sizes and volumes, pore size distributions, and the synthesis procedure and amounts of bentonite. Figure 3 presents the experimental isotherms for the SBB1, SBB2, and SBB3 samples, and the isotherms for the selected materials from the second group (BTS1, BTS5, and BTS9). In all cases, the reversible isotherms and hysteresis loops between the adsorption and desorption branches were observed. For samples with a standard amount of TEOS, type IV isotherms with the H1 hysteresis loop were obtained. Hysteresis is located in a similar pressure range, but the isotherms differ in the level of gas adsorption related to the specified surface area (S_BET_). The highest and lowest values of S_BET_ for the samples SBB1 and SBB3 were obtained, respectively. Similarly, the specific surface area for the BTS1 sample was slightly higher than for BTS9. Table 1 shows the textural properties of selected materials.

It was noted that the total specific surface area of the materials decreases in both synthesis groups with increasing bentonite content, however, even for the BTS9 sample (synthesized without TEOS), the S_BET_ value is still significant. For comparison, the S_BET_ of pure bentonite was very low and equals ~57 m^2^/g. A similar trend of changes applies to both the surface area of micropores and the pore volume of the SBB1-SBB3 samples. In the second group of materials, the surface area of micropores decreases with the increase of bentonite content. For the two first samples (BTS1 and BTS2), higher values of the total specific surface area, pore volume, and pore size were noticed in comparison to SBB3 (with a standard amount of TEOS used).

**Figure 4:**
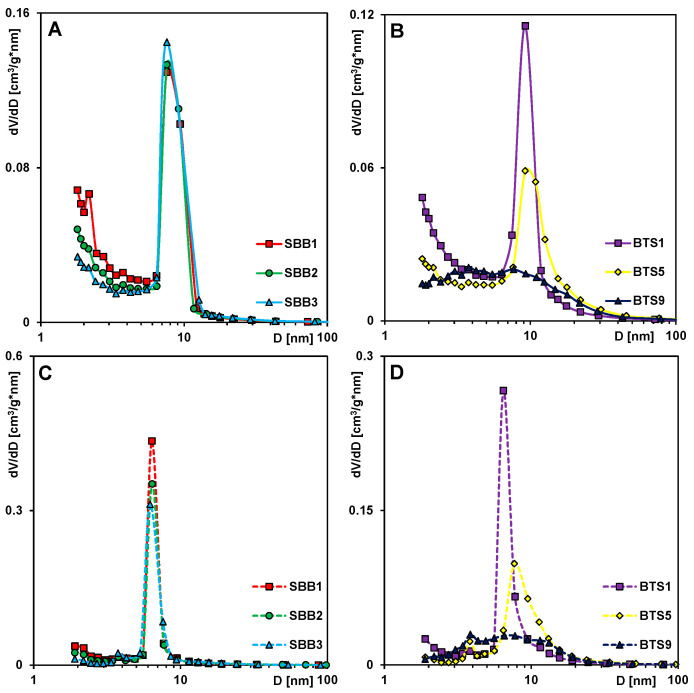
Pore size distributions calculated using the Barrett, Joyner, and Halenda BJH method for adsorption (**A**,**B**) and desorption (**C**,**D**) adsorption data.

The pore size distributions calculated from adsorption and desorption data are presented in Figure 4, and the pore diameters D_BJH_ are given in Table 1. One can find that the pores of the investigated materials are localized in the mesopore range, and the average pore size changes from 5.64 nm to 6.96 nm for the SBB1–SBB3 group of materials and from 6.2 nm to 11.25 nm for the second group of samples BTS1–BTS9. Maxima of the PSDs obtained from adsorption data are located at ~8 nm for SBB1, SBB2, and SBB3, however, PSDs calculated from desorption data reveal similar sizes of pores, although the peak for the SBB3 sample is shifted towards a lower value (about 6 nm). When the amount of TEOS decreases (the amount of bentonite increases), one can observe a widening of pore size distributions, which indicates an increase in structural heterogeneity.

### 2.3. Characterization of the Materials by Scanning-, Transmission Electron Microscopy and X-ray Fluorescence Technique

**Figure 5:**
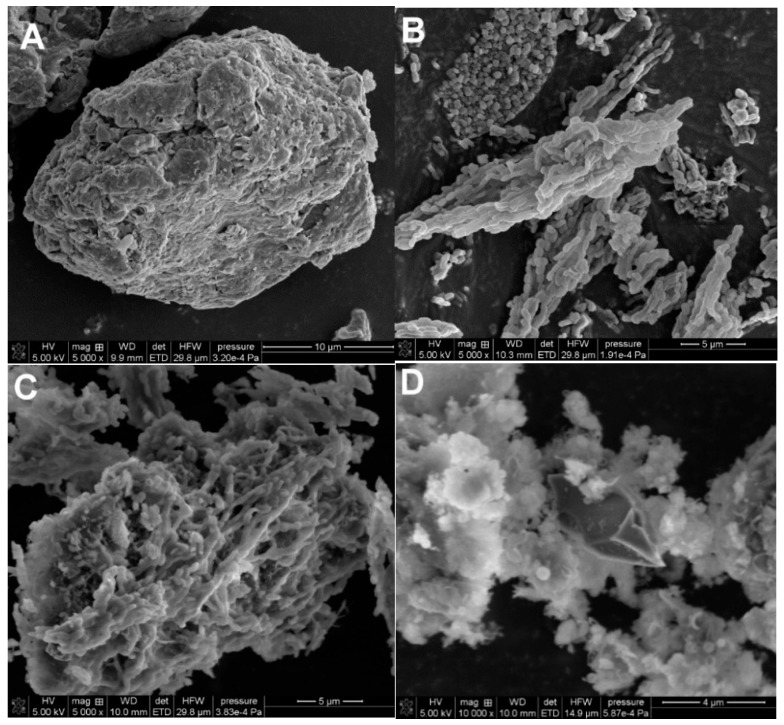
SEM images of selected composite materials (**A**) BN, (**B**) SBB3, (**C**) BTS1, and (**D**) BTS9.

Figure 5 shows the electron microscopy images for the selected samples (BN, SBB3, BTS1, BTS9). The scanning electron microscopy images show the general morphology and particle characteristics of air-dried composites. The irregular shape and chaotic general arrangement of granules and particles of the studied materials are observed. Some well-dispersed particles are stuck together forming the typical fluffy structure of aluminosilicate composites. It can be seen that the morphology of the SBB3 and BTS1 samples represented by structures of elongated sticks is almost the same as those observed for the SBA-15 material. In turn, BTS9 is characterized by a completely different morphology that is in good agreement with the results of the X-ray diffraction studies, indicating the amorphous, and disordered structure of this sample. Additionally, a pure BN sample for comparison is imaged.

**Figure 6:**
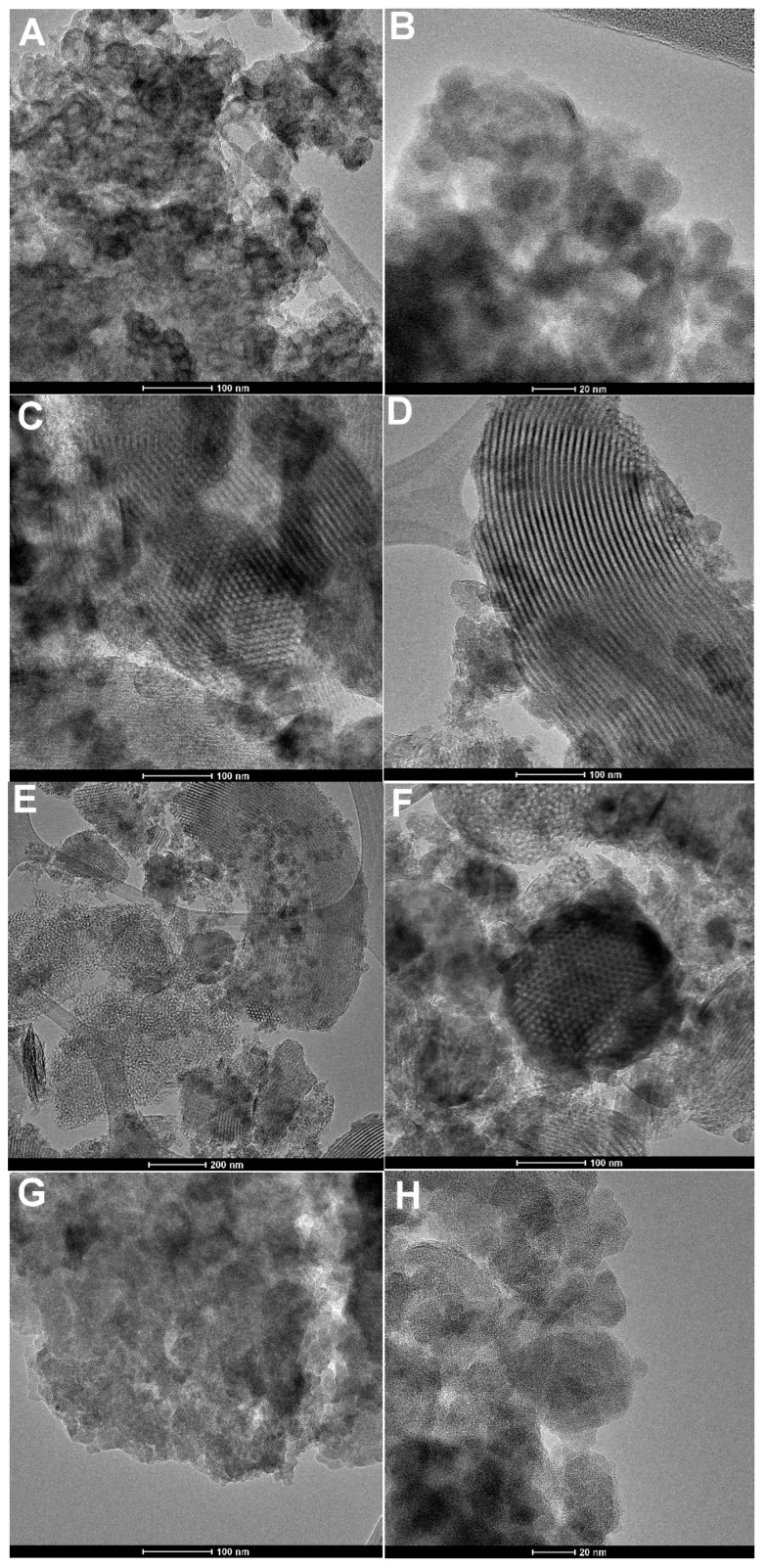
TEM images of selected composite materials:(**A**,**B**) BN, (**C**,**D**) SBB3, (**E**,**F**) BTS1, and (**G**,**H**) BTS9.

TEM images (Figure 6) suggest the successful synthesis of the nanocomposites with like-SBA-15 form, while EDX data shows a significant change in Al concentration for the BTS9 sample in comparison to BTS1. Table 2 collects the contribution of respective elements determined by TEM/EDX. As can be seen, the content of aluminum in the investigated materials depends on the bentonite amount. The TEM analysis reveals the well-ordered pore structure of 2 investigated samples (visible pores ordering for SBB3 and BTS1) and its lack of BN and BTS9 samples. The hexagonal pore structure and the parallel arrangement of the channels are noticeable for the samples where the hexagonal symmetry was previously noticeable by SAXS. The morphology changes observed microscopically clearly depend on the amount of bentonite added. For the SBB3 sample (TEOS/BN = 3.1), the significant areas with ordered characteristics are observed, while for the BTS9 sample (TEOS/BN = 2.7), additional fragments of the amorphous structure are visible between the ordered areas.

In Table 3, contribution of the respective components for three selected composites determined by the XRF technique was collected. One can see that composition of the composites depends on the quantitative content of reagents used in the synthesis process. A gradual increase in the bentonite amount in a reaction mixture (1.5; 2.5 and 5.5 g for the BTS1; BTS3 and BTS9 composites, respectively) results in a systematic increase in the content of components, such as alumina, iron oxide, and calcium oxide, in the final materials. In parallel, a decrease of silica related to lowering the TEOS amount as a reagent is observed. Generally, changes in the silica content in the composites with extremely different composition characteristics are not significant, while the alumina content increases nearly threefold. Nevertheless, the contribution of the latter component is incomparably small in relation to silica.

### 2.4. Potentiometric Titration Measurements for pHpzc Determination

**Figure 7:**
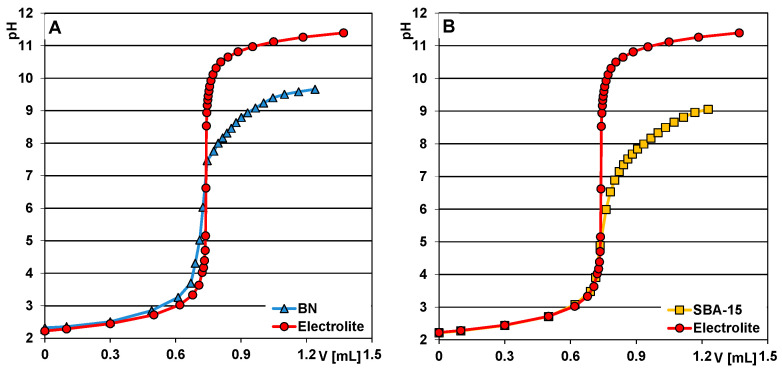

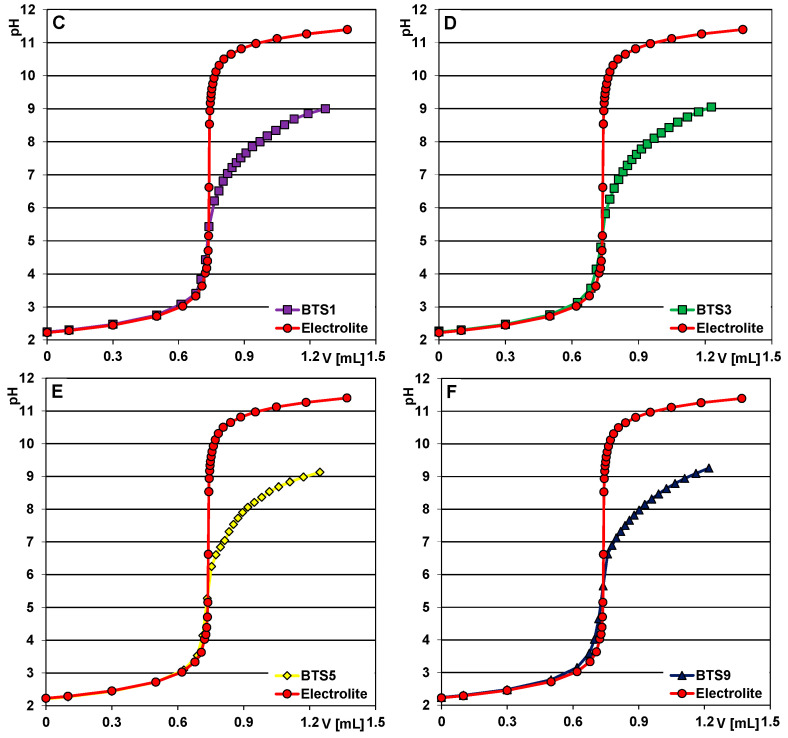
Dependences of suspension pH on titrant volume for (**A**) BN, (**B**) SBA-15, (**C**) BTS1, (**D**) BTS3, (**E**) BTS5) and (**F**) BTS9.

In Figure 7, the dependences of suspension pH on titrant volume for the selected materials measured by potentiometric titration are presented. The values of pH_PZC_ (point of zero charge) were estimated from the intercept of the titration curves for electrolyte and suspension. This parameter equals 7.1; 5.0; 5.1; 5.2; 5.5 and 5.9 for bentonite; SBA-15; BTS1; BTS3; BTS5 and BTS9, respectively. One can see that replacement of TEOS with a gradually increasing amount of bentonite in the synthesis procedure resulted in a shifting of the pHpzc values towards higher values. The discussed parameter corresponds to solution pH at which all positively and negatively charged surface moieties of solid are in a mutual balance. At pH conditions above pH_pzc_, a solid framework is negatively charged, while below pH_pzc_ is positively charged. The observed variations of acid–base properties of the studied materials are related to ionization of surface functional moieties i.e., (i) silanol, silanediol, silanetriol; (ii) hydrous oxides (bound with metals). The surface charge of bentonite and composites depends on the electrical state of all those groups. In the case of pure silica, the surface charge is derived only from moieties mentioned in bullet point (i). In our experiment, alterations in the composition of the substrate applied during the synthesis of the respective composites affected the chemical nature of their surfaces, visible in the pH_pzc_ shift by nearly one unit (for composites with the most different acid–base properties).

### 2.5. Adsorption of Thorium(IV) Ions from Aqueous Solutions on the Composites

In addition to the comprehensive morphological, textural, and structural characteristics of the obtained materials, studies in terms of their application in environmental protection were undertaken. For this purpose, the selected composites were used as adsorbents in the adsorption experiment of removing thorium(IV) ions from aqueous solutions. Thorium(IV) was treated in the study in two ways: (i) as a model substance concerning its chemical similarity with other more toxic tetravalent actinides; (ii) as an actual pollutant of the natural environment in the form of gaseous, liquid, and solid wastes produced by the mining and electrotechnical industries [59,60,61].

The results of adsorption studies for the composites are collected in Table 4. For comparative purposes, the tests comprised also pure SBA-15 silica and bentonite.

The thorium adsorption amounts obtained for the investigated composites are higher (22.05–23.07 mg/g) or near (20.05 mg/g) than for pure SBA-15 silica (20.65 mg/g). In the case of pure bentonite, its adsorption effectiveness towards thorium is the lowest (7.4 mg/g). To explain the differences in the adsorption capability of the materials, the structure characteristics and mechanism of the adsorption process are considered. It was assumed, that the main adsorption mechanism in the experimental systems was based on the electrostatic attraction forces between positively charged metal species and the negatively charged hydroxyl groups of solid surfaces. The possible forms of surface hydroxyl groups were as follows: silanol, silanediol, silanetriol (silica/bentonite/composites), or hydrous oxide (bentonite/composites). According to Weijuan [62], the adsorption potential of hydrous oxide groups towards thorium is greater compared to other surface moieties, mentioned above. Besides the type and number of active sites, of importance is their accessibility to the pollutant. The high porosity of the solid is a guarantee of successful access to the surface functional groups which are located in the material framework. The performance of adsorbents is also affected by the pH conditions of the experiment due to the type of assumed adsorption mechanism. As results from the potentiometric titration studies, the values of pHpzc of the SBA-15 and composites were equal to or above five, which means that adsorption surface sites were predominantly in the deprotonated form, favoring the adsorption process.

Analyzing the adsorption data along with textural parameters and composition of the studied materials, one can conclude that adding bentonite as a silica precursor is an effective way of obtaining low-cost and efficient adsorbent. Despite the worsening of global structure characteristics with an increase of bentonite amount to the reaction mixture, and simultaneous deterioration of the textural characteristics and loss of pore ordering, the adsorption properties are kept for the series of materials BTS1–BTS9. As a crucial factor in thorium(IV) adsorption, one can assume the accessibility of adsorbate to active sites of adsorbents. When elevating the bentonite amount as a synthesis substrate, the increase of Al_2_O_3_ content in the structure of novel materials should be highlighted. A high affinity of thorium ions towards alumina was reported previously in the literature [63]. In the case of pure bentonite with poor porosity, the result was three times worse in the removal of pollutants from aqueous solution compared to the effectiveness of new composites (despite the significant Al_2_O_3_ contribution in total bentonite composition). The composite BTS9 which was synthesized using only bentonite as a silica source is characterized by much better porosity which translates into greater adsorption effectiveness. Moreover, it should be underlined that in comparison to other composites with better porosity characteristics (BTS1–BTS8) its adsorption capability is on a similar level.

**Figure 8:**
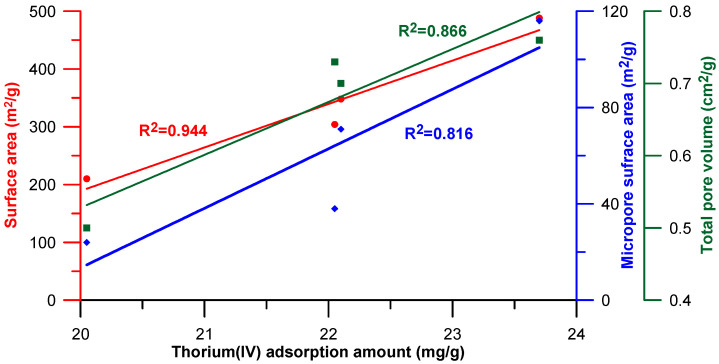
Dependence of thorium adsorption value for the selected composites on their textural characteristics.

The above conclusions are confirmed by strict correlations between the thorium(IV) adsorption values and the structural parameters of the composites, i.e., (i) specific surface area; (ii) micropore surface area; (iii) total pore volume which is presented in the graphical form in Figure 8. The determination coefficients for the investigated dependences ranged from 0.816–0.944.

A comparative study on the adsorption capacity for various materials reported in the literature (Table 5) indicated that the prepared bentonite-based composites may be successfully used in the adsorption removal of thorium(IV) and other actinides from aqueous solutions.

## 3. Materials and Methods

### 3.1. Materials and Chemicals

Self-assembly agent Pluronic P123 is composed of blocks of different polymerized monomers (poly(ethylene oxide)–poly(propylene oxide)–poly(ethylene oxide) (EO_20_PO_70_EO_20_, Mw = 5800), hydrochloric acid HCl (concentration ~38%) and tetraethyl orthosilicate (TEOS, 98%) as an organic silica source were purchased from Sigma-Aldrich. All solutions were prepared with deionized water, and reagents were used as supplied, without further purification. Pure bentonite clay with the composition (in wt%)–59.2% SiO_2_, 17.5% Al_2_O_3_, 5.61% Fe_2_O_3_, 4.11% CaO, 3.34% Na_2_O, 1.99% MgO, and 1.3% K_2_O was purchased from Sigma-Aldrich, Gillingham, UK.

### 3.2. Synthesis of the Ordered Mesoporous Materials

In detail, two groups of materials were obtained by two different approaches (Table 6). In the first case, three samples containing a standard amount of TEOS (4.65 mL) and different amounts of bentonite (0.5 g, 1 g, and 1.5 g) were performed according to a similar route to [73]. In the second case, the amount of TEOS was reduced from 4.15 mL until the TEOS reagent was eliminated and replaced by bentonite. In turn, the amount of bentonite was increased from 1.5 g to 5.5 g.

Next, the mixtures were stirred for 24 h at 40 °C. The samples were then transferred to a vacuum dryer, where for 48 h at a temperature of 100 °C, the aging process of the mixtures was carried out. The samples were then filtered and calcined at 550 °C for 5 h for removing the organic phase and opening the porous structures. Table 6 shows the experimental details and the amounts of chemicals used during synthesis.

### 3.3. Adsorption of Thorium(IV)

The adsorption of thorium(IV) was carried out as follows: 0.2 g sample of the composite was contacted with 50 mL of Th(NO_3_)_4_ at the concentration of 5 × 10^−1^ mmol/dm^3^. The mixture was kept in a thermostatic shaker Innova 40R Model (25 °C, 110 rpm) (New Brunswick Scientific, Enfield, CT, USA) for 48 h. The amount of adsorbed thorium ions was assessed by the XRF technique from the solution phase, as the loss of element in the solution resulted from the adsorption process. Parallel, the calibration curve of solutions with different concentrations of thorium ions was determined. XRF estimations were performed using the PANalytical algorithm.

### 3.4. Measurements and Calculations

The obtained materials were analyzed by X-ray diffraction (XRD) using an Empyrean diffractometer (PANalytical, Malvern, UK) with CuKα radiation (λ = 1.5418 Å) in the wide range of 2θ (2–90 degrees of 2θ). The XRD analysis system was equipped with a Cu LFF HR X-ray tube with generator settings of 40 kV and 30 mA during analysis. The wide-angle XRD analysis was performed using a reflection-transmission spinner suitable for powders and solid objects in reflection geometry. The SAXS analysis was realized with the same equipment using SAXS/WAXS stage. The SAXS configuration included 0.2–5 degrees of 2θ, generator settings of 40 kV and 40 mA, and reflection geometry. Evaluation of the porosity of the composite samples was performed using a low-temperature isothermal nitrogen adsorption–desorption using an automatic ASAP2020 device (Micromeritics, Norcross, GA, USA). Specific surface areas were computed from experimental isotherms by applying the BET theory and linear range of the BET plot [74]. Pore size distribution functions (PSD) were calculated using the Barrett, Joyner, and Halenda (BJH) procedure. All samples were outgassed before analysis at 100 °C for 24 h in the degassing port of the analyzer. The dead space volume was measured for calibration on experimental measurement using helium as an adsorbate. Transmission electron microscopy with X-ray microanalysis (TEM/EDX) was carried out on a high-resolution transmission electron microscope Tecnai 60–200 (FEI Company, Washington County, OR, USA) operating at 200 kV. The scanning electron microscope Quanta 3D FEG by FEI was used to make the SEM microphotograph imaging. The composition characteristics of the materials was made based on measurements by X-ray Fluorescence Technique (spectrometer Axios mAX, PANalytical, Malvern, UK). The values of pH_PZC_ of the materials were determined from the potentiometric titration measurements with NaOH solution. In the measurement procedure, electrolyte (NaCl) with ionic strength of I = 0.1 mol/dm^3^ and hydrochloric acid solution (to acidify suspension) were used.

## 4. Conclusions

This paper presents a structural, textural, and morphological characterization of novel mesoporous materials synthesized using aluminum phyllosilicate clay (bentonite) as an untypical silica source. In this work, the possibility of applying natural aluminosilicate as a counterfactual source of silica for the preparation of mesoporous silicates was proposed and optimized. The great advantage of aluminum phyllosilicate clay (bentonite) is cost reduction, availability, and environmental friendliness. Collected data suggest that even after the complete elimination of TEOS and using bentonite as a silica source, porous materials were obtained. The results indicate that bentonite is a good potential silica replacement agent, however, its application may reduce the pore ordering of final composites. In addition, the quantitative analysis of the obtained materials indicates the appropriate way of incorporating the aluminum and other heteroatoms from mineral structure to mesoporous silicate scaffold, which undoubtedly can improve the sorption capacity of mesoporous silica materials. Finally, this study proposes a new, cost-effective and novel composite for the removal of Th(IV) and may be promising for eliminating other heavy metal ions from the environment. Thus, the synergistic effect of expanding the porous surface (using bentonite as a silica precursor) and the presence of thorium-binding groups (such as Al_2_O_3_) has been emphasized. The synthesized composites show sorption capacity for Th(IV) with a maximum of 23.7 mg/g and seem to be good materials in comparison to other adsorbents described in the literature.

## Figures and Tables

**Table 1:** Textural properties of investigated composites.

Sample	^a^ S_BET_ (m^2^/g)	^b^ S_mic_ (m^2^/g)	^c^ V_t_ (cm^3^/g)	^d^ V_mic_ (t-plot) (cm^3^/g)	^e^ D_BJH_ (nm)
SBA-15	538	35	0.62	0.013	4.60
BN	57	21	0.09	0.009	6.59
SBB1	611	113	0.86	0.0458	5.64
SBB2	500	106	0.74	0.0442	5.94
SBB3	411	95	0.61	0.0405	5.96
BTS1	488	116	0.76	0.049	6.2
BTS2	419	123	0.68	0.054	6.5
BTS3	348	71	0.70	0.029	8.1
BTS4	339	64	0.69	0.026	8.19
BTS5	304	38	0.73	0.016	9.63
BTS6	312	30	0.87	0.011	11.13
BTS7	268	27	0.75	0.01	11.24
BTS8	210	15	0.59	0.005	11.25
BTS9	210	24	0.50	0.010	9.44

^a^ BET surface area, calculated using experimental points at relative pressures of (P/P_o_) 0.035–0.31, where P and P_o_ denote the equilibrium and saturation pressures of nitrogen, respectively; ^b^ Specific surface area of micropores calculated using t-plot, ^c^ Total pore volume calculated from the nitrogen volume adsorbed at P/P_o_ = 0.99; ^d^ Pore volume of micropores determined by t-plot, ^e^ Pore-size distribution curves were obtained from the adsorption branch using the Barrett–Joyner–Halenda (BJH) model with cylindrical pores.

**Table 2:** The composition of the selected composites (TEM/EDX).

Sample	SBB3		BTS1		BTS9	
	% Weight	% Atomic	% Weight	% Atomic	% Weight	% Atomic
C	3.01	4.96	1.50	2.51	0.59	1.66
O	50.42	62.39	50.23	63.04	51.12	63.96
Mg	0.17	0.14	-	-	0.34	0.28
Al	1.29	0.94	0.93	0.69	3.95	3.19
Si	44.21	31.16	47.03	33.63	42.47	30.27
K	0.28	0.14	-	-	0.30	0.15
Ca	0.16	0.08	-	-	0.17	0.08
Fe	0.43	0.15	0.28	0.10	1.02	0.36

**Table 3:** Contribution of the respective components for the selected composites (XRF analysis), and quantitative content of reagents used in their synthesis process.

Sample	%SiO_2_	%Al_2_O_3_	%Fe_2_O_3_	%CaO	TEOS/BN (mL/g)
BTS1	98.5	1.3	0.12	0.08	4.15/1.5
BTS3	97.8	1.8	0.23	0.17	3.15/2.5
BTS9	95.5	3.8	0.42	0.28	0/5.5

**Table 4:** The adsorption effectiveness of the selected materials towards thorium(IV) ions and their textural characteristics.

Sample	Adsorption Amount (mg/g)	S_BET_/S_mic_/V_t_ (m^2^/g)/(cm^3^/g)	pH_pzc_
Bentonite	7.4	57/21/0.09	7.1
SBA-15	20.65	538/35/0.62	5.0
BTS1	23.7	488/116/0.76	5.1
BTS3	22.1	348/71/0.70	5.2
BTS5	22.05	304/38/0.73	5.5
BTS9	20.05	210/24/0.50	5.9

**Table 5:** The adsorption capacity of various materials towards thorium(IV).

Adsorbent	Adsorption Capacity (mg/g)	Reference
MCF/NaY based foam	23.7	[63]
Fibrous radiation grafted material	24.9	[64]
Acid modified bentonite	14.3	[65]
Insolubilized humic acid	20	[66]
Illite	7.2	[67]
Na-Bentonite	11.4	[68]
Diatomite	30.3	[69]
Modified powdered waste sludge	27	[70]
Sulfated β-cyclodextrin inclusion complex	12.7	[71]
Modified polyrotaxane	12.9	[72]
BTS1	23.7	this paper

**Table 6:** Experimental details and description of the mesoporous aluminosilicate composites.

Group I				Group II			
Sample	Pluronic P123 (g)	TEOS (mL)	Bentonite (g)	Sample	Pluronic P123 (g)	TEOS (mL)	Bentonite (g)
SBB1	2	4.65	0.5	BTS1	2	4.15	1.5
				BTS2		3.65	2.0
				BTS3		3.15	2.5
SBB2		4.65	1.0	BTS4		2.65	3.0
				BTS5		2.15	3.5
				BTS6		1.65	4.0
SBB3		4.65	1.5	BTS7		1.15	4.5
				BTS8		0.65	5.0
				BTS9		0.00	5.5

## Data Availability

The data are available by the corresponding author.

## Sample Availability

Samples of the composites available from the authors.
